# Comparison of CRP, Procalcitonin, Neutrophil Counts, Eosinophil Counts, sTREM-1, and OPN between Pneumonic and Nonpneumonic Exacerbations in COPD Patients

**DOI:** 10.1155/2022/7609083

**Published:** 2022-03-31

**Authors:** Shan Mou, Wei Zhang, Yan Deng, Zhijun Tang, Depeng Jiang

**Affiliations:** ^1^Department of Respiratory Medicine, the Second Clinical Hospital of Chongqing Medical University, Chongqing, China; ^2^Department of Respiratory Medicine, The People's Hospital of Nanchuan, Chongqing, China

## Abstract

**Introduction:**

The patients with community-acquired pneumonia (CAP) and acute exacerbations of COPD (AECOPD) could have a higher risk of acute and severe respiratory illness than those without CAP in AECOPD. Consequently, early identification of pneumonia in AECOPD is quite important. Methods. 52 subjects with AECOPD + CAP and 93 subjects with AECOPD from two clinical centers were enrolled in this prospective observational study. The values of osteopontin (OPN), soluble triggering receptor expressed on myeloid cells-1 (sTREM-1), C-reactive protein (CRP), procalcitonin (PCT), eosinophil (EOS) counts, and neutrophil (Neu) counts in blood on the first day of admission and clinical symptoms were compared in AECOPD and AECOPD + CAP. In addition, subgroup analyses of biomarker difference were conducted based on the current use of inhaled glucocorticoids (ICS) or systemic corticosteroids (SCS).

**Results:**

Patients with AECOPD + CAP had increased sputum volume, sputum purulence, diabetes mellitus, and longer hospital stays than AECOPD patients (*p* < 0.05). A clinical logistic regression model showed among the common clinical symptoms, purulent sputum can independently predict pneumonia in AECOPD patients after adjusting for a history of diabetes. At day 1, AECOPD + CAP patients had higher values of Neu, CRP, PCT, and OPN, while serum sTREM-1 levels and EOS counts were similar in the two groups. CRP fared best at predicting AECOPD with CAP (*p* < 0.05 for the test of difference), while OPN had similar accuracy with Neu, PCT, and purulent sputum (*p* > 0.05 for the test of difference). Multivariate analysis, including clinical symptoms and biomarkers, suggested that CRP ≥15.8 mg/dL at day 1 was a only promising predictor of pneumonia in AECOPD. CRP and OPN were not affected by ICS or SCS.

**Conclusions:**

CRP ≥15.8 mg/dL is an ideal promising predictor of pneumonia in AECOPD, and its plasma level is not affected by ICS or SCS. The diagnostic performance of CRP is not significantly improved when combined with clinical symptoms or other markers (OPN, PCT, and Neu).

## 1. Introduction

Chronic obstructive pulmonary disease (COPD) is characterized by persistent and progressive respiratory symptoms and airflow limitation due to inhalation of noxious gases most commonly from tobacco. Acute exacerbation of COPD (AECOPD) is a sudden and sustained worsening of respiratory symptoms in the natural course of COPD requiring additional therapy. Many triggers have been shown to incite AECOPD, among which infectious origin, including viral, bacterial, or both pathogens, is the commonest inciting cause for AECOPD [1, 2]. These cases admitted with community-acquired pneumonia (CAP) were diagnosed with AECOPD + CAP. According to this study, the patients who present with radiological pneumonia could have a more severe illness and a poorer prognosis than did those admitted without pneumonia in exacerbation of COPD [3–5]. Consequently, early identification of patients with AECOPD and pneumonia is crucial for outcome. However, it is not easy to make an early diagnosis of pneumonia due to an blurred clinical picture or interpretation of the chest film. Hence, in order to initiate the proper treatments timely and improve their prognosis, clinicians need reliable surrogate biomarkers along with clinical signs and symptoms to predict pneumonia in patients with AECOPD.

Osteopontin (OPN) is expressed in plenty of immune cells, including neutrophils, macrophages, dendritic cells, natural killer cells, and T cells. It regulates both innate and adaptive immune responses by mediating Th1 inflammation, neutrophil chemotaxis, and matrix remodeling [6]. OPN has been found to be associated with lung inflammation in different pulmonary diseases, including stable COPD [7, 8], AECOPD [9], *Klebsiella pneumoniae*-induced pneumonia [10], and pneumococcal pneumonia [11]. However, less is known about serum OPN levels in admitted AECOPD patients with pneumonia. OPN may also be a useful biomarker to discriminate pneumonia from AECOPD.

A triggering receptor expressed on myeloid cells (TREM-1), as a receptor, is expressed on neutrophils and monocytes. Its expression is upregulated on phagocytic cells by bacterial or fungal products [12, 13]. sTREM-1 is a soluble form of TREM-1. Previous studies indicated that sTREM-1 is a diagnostic marker for the presence of infectious disease, including pneumonia [14] and sepsis [15, 16]. In addition, the serum level of sTREM-1 is also increased in many noninfectious respiratory diseases, such as asthma and AECOPD [17, 18]. Hence, sTREM-1 may be useful for AECOPD and pneumonia. However, the value of serum sTREM-1 in distinguishing among pneumonic and nonpneumonic exacerbations in COPD patients has not been evaluated.

In summary, elevated levels of the abovementioned biomarkers seem to be implicated in the pathogenesis of pneumonia and AECOPD. Besides, neutrophil (Neu) counts, C-reactive protein (CRP), and procalcitonin (PCT) in peripheral blood are increased in AECOPD patients and more so in AECOPD complicated with pneumonia [19–22]. Lower eosinophil (EOS) counts have been reported in patients with COPD + CAP compared to AECOPD [21]. Consequently, in this prospective observational study, we compared the differences of CRP, PCT, OPN, sTREM-1, Neu, and EOS and clinical parameters in AECOPD patients with or without CAP in China. In addition, we compared with the diagnostic profiles of the markers between pneumonic and nonpneumonic exacerbations in COPD patients. Meanwhile, subgroup analyses of biomarker difference were conducted based on the current treatment of inhaled glucocorticoids (ICS) or systemic corticosteroids (SCS).

### 1.1. Patients and Methods

The prospective study followed the declaration of Helsinki and was conducted from November 2016 to February 2019 after receiving the approvals from the Ethics committee of the Second Clinical Hospital of Chongqing Medical University and the People's Hospital of Nanchuan, Chongqing. All subjects signed informed consent before inclusion in this study. All patients who had a history of COPD confirmed by spirometry and were clinically identified with AECOPD were recruited in this study once admitted to hospital. AECOPD is characterized by sudden worsening of respiratory symptoms, including increased dyspnea, sputum volume, and sputum purulence. CAP was defined according to the following criteria: (1) clinical signs and symptoms of lung infection as well as exudations or opacities on a chest computed tomograph at admission and (2) compatible symptoms occur in the community rather than in the hospital. The CURB-65 pneumonia severity score was assessed, and its interpretation is as follows: 0–1: probably suitable for home treatment, low risk of death; 2: consider hospital supervised treatment; and 3: manage in the hospital as severe pneumonia, high risk of death. Exclusion criteria were as follows: ① other significant respiratory diseases, including asthma, tuberculosis, bronchiectasis, and pulmonary fibrosis; ② ischemic heart disease, such as a history of coronary heart disease, acute heart failure or acute attack of chronic heart failure, and acute myocardial infarction; ③ malignancy; ④ autoimmune diseases; ⑤ HIV infection; ⑥ patients who had hospitalized in the previous 2 weeks; and ⑦ other inflammatory conditions or inflammatory processes that could be associated with abnormal sTREM-1, OPN, PCT, Neu, EOS, and CRP levels. In all subjects, plasma CRP, Neu, EOS, and plasma PCT were measured by routine automated tests on admission. Data on demographic variables were collected on admission. Clinical manifestations of acute episodes were recorded, and data on clinical status during hospitalization were collected.

### 1.2. Sample Collection and Measurement of OPN and sTREM-1

Blood samples were drawn in coagulation promoting tubes and centrifuged at 3000 rpm for 10 min at room temperature within 120 minutes of collection. The supernatants were obtained and stored at −80°C until analysis; the serum fraction was withdrawn and stored at −80°C for measurements of OPN and sTREM-1 using enzyme-linked immunosorbent assay (ELISA) kits (CUSABIO, Wuhan, China). The laboratory team who determined OPN and sTREM-1 was blinded to the clinical data.

### 1.3. Statistics

Normally distributed variables were presented as mean ± standard deviation (SD), and the independent two-tailed test was used to analyze differences between the two groups. Skewed variables were presented as median (interquartile ranges, IQR), and differences in the variables were analyzed using the Mann–Whitney *U*-test. Categorical variables are described as frequencies and proportions, and their differences were evaluated by the X2 test or Fisher's exact test when necessary. Variables with a *p* < 0.10 in the univariate analysis were retained as covariates in the forward stepwise selection procedure of multivariate analyses. A Hosmer–Lemeshow goodness-of-fit test was also figured up. The abovementioned statistical analyses were conducted using the SPSS 23 software program (IBM Corporation, Armonk, NY, USA), and *p* < 0.05 was regarded as statistical significance. The receiver operating characteristic (ROC) curves were calculated to compare the accuracy of various diagnostic tests in the diagnosis of pneumonia in patients with AECOPD using MedCalc software 15.8 (Mariakerke, Belgium).

## 2. Results

From November 2016 to February 2019, a total of 145 patients who met the inclusion criteria were enrolled, of whom 93 patients had AECOPD and 52 patients were diagnosed with AECOPD and CAP. The CURB-65 score was 0–2 in all patients. There were no significant differences between the two groups in terms of age, BMI, sex, current smoking history, percentage of patients treated with ICS, SCS, or antibiotics, and lung function (Table 1). Compared with AECOPD group, more patients in the AECOPD + CAP group had increased sputum volume, sputum purulence, diabetes mellitus, longer hospital stays than AECOPD patients (Tables 1 and 2). No significant difference was found in the proportion of patients requiring invasive/noninvasive mechanical ventilation, emergency treatment, and admission to the intensive care unit (Table 2).

**Figure 1:**
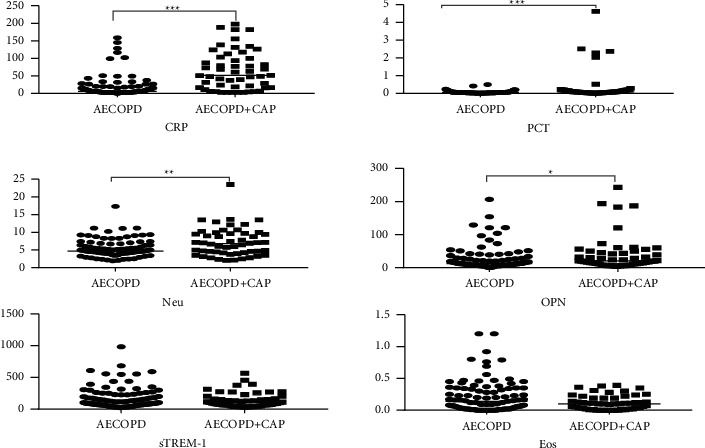
The levels of CRP, PCT, Neu, OPN, sTREM-1, and EOS in patients with AECOPD and AECOPD + CAP. Note: ^*∗*^*p* < 0.05; ^*∗∗*^*p* < 0.01; ^*∗∗∗*^*p* < 0.001.

**Figure 2:**
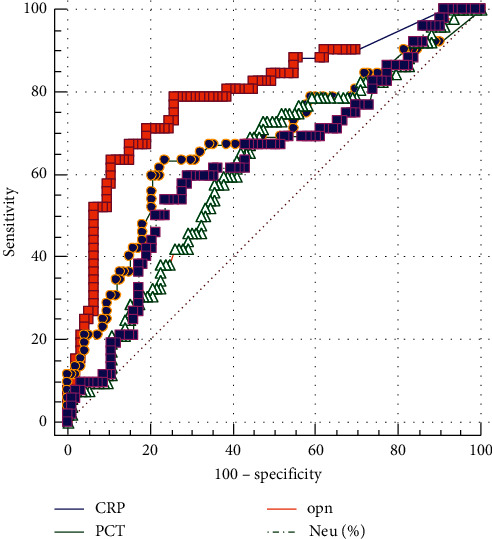
Receiver operating characteristic (ROC) curves of CRP, PCT, Neu, and OPN for differentiating between pneumonic and nonpneumonic exacerbations in AECOPD patients.

On the first day after admission, the levels of CRP, PCT, Neu, and OPN in AECOPD + CAP patients were significantly higher than those in AECOPD patients. However, there were no significant differences in serum levels of sTREM-1 and blood eosinophil counts between the two groups (Table 2, Figure 1). We found that CRP showed the best diagnostic performance, while PCT, Neu, and OPN presented similar diagnostic discrimination property. The area under the ROC curve (AUC) was 0.797 (95% CI 0.72–0.88) for CRP, 0.678 (95% CI 0.58–0.77) for PCT, 0.63 (95% CI 0.53–0.73) for Neu, and 0.622 (95% CI 0.53–0.72) for OPN (Table 3, Figure 2). A clinical logistic regression model, including common clinical symptoms, showed an increased risk of CAP in AECOPD patients with increased sputum purulence compared to those without increased sputum purulence after adjusting for a confounding factor—a history of diabetes (OR = 2.39, 95% CI 1.17–4.87) (Table 4). The AUC value of purulent sputum was 0.62 (95% CI 0.52–0.71). Its diagnostic accuracy was comparable to that of OPN, Neu, and PCT (*p*=0.92,  0.85,  and 0.33, respectively for the test difference), but significantly lower than that of CRP (*p*=0.0009 for the test difference). To better characterize early clinical predictors of pneumonia in AECOPD patients, another logistic regression analysis (Table 5) was performed. Variables, including sputum purulence, increased sputum volume, diabetes mellitus, FEV1, % predicted, PCT ≥ 0.07 ng/mL, Neu ≥ 6.41 × 10^9/L, CRP ≥ 15.8 mg/dL, OPN ≥ 13.26 ng/mL, and SCS treatment, were retained as covariates in the forward stepwise selection procedure of multivariate analysis. We found that the plasma levels of CRP ≥ 15.8 mg/dL are independent predictive factors of pneumonia in AECOPD.

We conducted subgroup analyses of biomarker difference in AECOPD patients with and without pneumonia. One based on the prior use of ICS, and the other on prior administration of SCS. In AECOPD patients with or without pneumonia, there were no significant differences in CRP, OPN, Neu, EOS, PCT, and sTREM-1 levels between patients being treated with ICS and those who were not (Table 6). However, some biomarker levels were affected by SCS treatment. We observed that neutrophil counts were significantly higher in patients who were being treated with SCS than those who were not, while AECOPD patients being treated with SCS had lower serum sTREM-1 levels and eosinophil counts compared to those who were not (Table 7).

## 3. Discussion

Patients with COPD are prone to CAP than those without COPD [23, 24]. Early identifying patients with AECOPD and CAP aids clinical treatment decisions, thus improving their prognosis. In clinical work, it is not easy to make an early diagnosis of pneumonia because sometimes chest X-ray is not sensitive enough for identifying pulmonary exudation and consolidation, compared to chest computed tomography [25–27]. Although chest CT has higher resolution, it sometimes cannot distinguish exactly between new active infections and chronic infections, resulting in unclear interpretation of the chest CT. In addition, more and more evidences suggested that the lower airway inflammation in AECOPD may correlated with the systemic inflammatory response [28]. Consequently, based on these, more and more scholars are trying to find reliable markers to predict pneumonia in AECOPD in recent years, thus helping guide clinicians to make correct treatment decisions in these patients. The present study demonstrated that ① the logistic regression model, including the common clinical symptoms except for biomarkers as covariates, showed only purulent sputum could predict AECOPD + CAP. ② CRP on day 1 was significantly better than PCT, OPN, Neu, and purulent sputum for discrimination of pneumonic and nonpneumonic exacerbations in AECOPD patients, and OPN had similar diagnostic accuracy with Neu, PCT, and purulent sputum, while serum sTREM-1 and EOS could not be used to distinguish the two diseases. ③ Two subgroup analyses of biomarker difference conducted based on the prior use of ICS or SCS showed that in AECOPD patients with or without pneumonia, there were no significant differences in CRP, OPN, Neu, EOS, PCT, and sTREM-1 levels between patients being treated with ICS and those who were not. However, neutrophil counts, eosinophil counts, and serum sTREM-1 levels were affected by systemic corticosteroids. ④ A logistic regression model, including biomarkers and clinical symptoms as covariates, showed that only CRP ≥ 15.8 mg/dL is an independent predictor of pneumonia in AECOPD.

There are still some controversies about the usage of biomarkers in the diagnosis of COPD with pneumonia. A study in 2013 comparing the diagnostic performance of CRP, PCT, TNF-alpha, IL-6 demonstrated that CRP at day 1 fared better than other biomarkers at identifying pneumonic exacerbations in COPD [22]. We found that CRP was significantly better than PCT, Neu, OPN, and purulent sputum in identifying CAP in AECPOD patients. However, two recent studies have shown that the AUC value of CRP was higher than PCT, and the test of difference was not statistically significant [19, 20]. Possible explanations for the gap may be due to the differences in COPD severity, pneumonia severity, and etiology. In addition, previous studies suggested that PCT seems to have a weak role in diagnosing bacterial infection during exacerbations of COPD with a low AUC value less than 70% [29, 30]. There are some controversies on the relationship between CRP and microbial etiology in people with acute exacerbations of COPD. Some people considered that CRP levels were higher in bacterial infections than in viral infections [31], but the others suggested that CRP may be associated with viral infections as well as mixed viral/bacterial infections of AECOPD [32]. Recent studies have shown that CRP and PCT might be of interest as biomarkers in guiding the use of antibiotics in patients with AECOPD, without evidence of harm [33, 34]. In this study, we take it that plasma CRP on day 1 which is not affected by the use of ICS or SCS is a promising biomarker to identify pneumonia in AECPD patients especially in some cases where the interpretation of the chest imaging was blurred.

COPD is predominantly characterized by TH1 cell-mediated neutrophilic airway inflammation which is often correlated with bacterial infections. Neutrophil in blood has been found to be increased in patients with AECOPD and pneumonia compared to AECOPD patients [21]. Gao et al reported elevated blood neutrophil counts in patients with pneumonia in COPD [21], consistent with the results of our trial. This finding needs to be confirmed by high-quality RCT studies in the future. Recently, more and more studies report that eosinophils are important in COPD. Blood eosinophil counts in COPD patients may be used to predict a beneficial responsive to corticosteroid and the risk of future acute exacerbations [1]. Studies comparing the difference of blood eosinophil counts between paitents with AECOPD with or without CAP were rare. A recent study showed that COPD + CAP patients had significantly lower eosinophil counts than those in AECOPD patients [21], which is in contradiction with our experimental results. We found no significant difference in eosinophil counts between patients AECOPD + CAP and AECOPD. There are two possible explanations for this contradiction: (1) the patients included in the trial might have different aetiologies from them; (2) some of the patients we enrolled had used glucocorticoids before the levels of blood inflammatory markers were measured because glucocorticoids can affect blood eosinophil counts.

Preclinical studies have shown that OPN seems to be involved in the pathogenesis of COPD and pneumonia. In our study, the increased serum level of OPN has been observed in patients with AECOPD and pneumonia, compared with patients with AECOPD. Future studies will have to explore the role of OPN in the pathogenesis of AECOPD and pneumonia and find its effective clinical applications, such as a possible treatment target. The diagnostic accuracy of OPN was similar to that of PCT and Neu, but significantly lower than that of CRP. AUC of serum OPN was 0.622 with a sensitivity of 73% and a specificity of 53% for diagnosing pneumonia in addition to AECOPD. Accordingly, serum OPN as a single biomarker was not so reliable for diagnosing AECOPD with pneumonia due to the low AUC value.

sTREM-1 was found to be as a promising diagnostic marker of the presence of pneumonia [14], sepsis [35], and AECOPD [18]. We discovered that sTREM-1, an established biomarker of systemic inflammation, showed no predictive value for differentiating pneumonic and nonpneumonic exacerbations in COPD patients. Similarly, in the study by Rohde et al., the value of serum sTREM-1 in the determination of AECOPD etiology was evaluated. The results showed that sTREM-1 could not be used to identify bacterial exacerbation of COPD [17]. In addition, our study found for the first time that serum sTREM-1 levels may be affected by systemic corticosteroids.

Systemic corticosteroids and inhaled glucocorticoids are often used in patients with AECOPD. We showed that the biomarker levels at day 1 were not affected by pretreatment with ICS, while sTREM-1, EOS, and Neu except PCT, OPN, and CRP were affected by SCS treatment. Studies have shown that corticosteroids can promote neutrophilia by mobilizing neutrophil reserve [36]. In addition, an animal study showed that glucocorticoids can cause eosinopenia through CXCR4-dependent migration of eosinophils to the bone marrow [37]. Therefore, we believe that short-term application of SCS can reduce blood eosinophil counts and increase blood neutrophil counts in AECOPD patients. To our knowledge, no study has explored the effect of glucocorticoids on sTREM-1 levels. We found that sTREM-1 levels in AECOPD patients may decrease after systemic application of corticosteroids. However, the mechanism involved is unknown.

Our study found that patients with AECOPD + CAP present more severe clinical parameters. The patients showed increased presence of sputum volume, sputum purulence, and diabetes mellitus compared with AECOPD patients. In agreement with these findings, studies comparing clinical features between pneumonic and nonpneumonic exacerbations in COPD patients confirmed patients with pneumonia in addition to AECOPD tend to show the presence of abnormal clinical symptoms and signs [4, 20, 22].

In our study, none of patients with AECOPD with/without pneumonia died, so we could not provide data on short-term mortality outcome for AECOPD + CAP vs AECOPD. There was no significant difference in the risk of respiratory failure, need for intensive care unit, invasive/noninvasive mechanical ventilation, and emergency treatment between the two groups except hospitalization days. The results indicated that short-term clinical outcomes during hospitalization were similar between groups.

## 4. Strengths and Limitations

Many previous studies have also compared the clinical and laboratory parameters of AECOPD and AECOPD + CAP. However, the vast majority of the studies used chest x-ray to diagnose pneumonia in AECOPD. In our study, chest computed tomography (CT) had been used to diagnose pneumonia, which could be more accurate. Another strength is that our study compare the clinical parameters and diagnostic performance of serum OPN, sTREM-1, CRP, PCT, EOS and Neu in a real-world environment. Consequently, these findings can be transferred to the hospital's outpatient clinics and emergency department.

There are some limitations in our study. One limitation is that we didn't follow up the patients after discharge. Another limitation is that we didn't know the levels of the biomarkers during stable COPD, and many inflammatory markers' levels in peripheral blood had been found to be increased during the stable phase of COPD. In addition, according to CRUB-65 score, pneumonia is not severe in patients included in our article. Therefore, our research results can only explain the relative difference between AECOPD patients with nonsevere pneumonia and those without pneumonia.

## 5. Conclusions

In summary, we find that AECOPD patients with pneumonia have more abnormal clinical features, similar short-term outcomes, and higher levels of inflammatory biomarkers, including CRP, PCT, OPN, and Neu but except sTREM-1 and EOS, compared to those with pneumonia. Among the common clinical symptoms, only purulent sputum can independently predict pneumonia in AECOPD patients. CRP by itself was more reliable than other laboratory values (PCT, OPN, Neu, and purulent sputum) in identifying pneumonia in AECOPD, independent of current treatment of ICS and SCS, indicating that CRP maintains the major role in this regard. Its diagnostic performance is not significantly improved when combined with clinical symptoms or other markers (OPN, PCT, and Neu).

## Figures and Tables

**Table 1:** Basic characteristics of subjects included.

	AECOPD patients	CAP + AECOPD patients	*p*
No.	93	52	
Age, years	72.6 ± 8.6	70.79 ± 9.5	0.20
BMI, kg m^−2^	22.53 ± 4.2	23.17 ± 3.3	0.41
Sex (male/female), n (%)	82 (88.2)/11 (11.8)	45 (86.5)/7 (13.5)	0.78
Current smokers, n (%)	41 (44.1)	17 (32.7)	0.18
Pharmacotherapy, n (%)			
ICS	85 (91.4)	47 (90.4)	1.0
SCS	31 (33.3)	25 (48.1)	0.08
Antibiotic	91 (97.8)	52 (100)	0.54
FEV1/FVC, n (%), median (IQR)	54.03 (43.8–61.7)	53.4 (41.4–61.02)	0.64
FEV1, % predicted, median (IQR)	49.4 (33.9–63.3)	45 (27.7–56.0)	0.08
COPD-GOLD stage I-II/III-IV, n (%)	48 (51.6)/45 (48.4)	34 (65.4)/18 (34.6)	0.11
CRUB-65 score 0/1/2, n (%)	11 (11.8)/54 (58.1)/28 (30.1)	10 (19.2)/28 (53.8)/14 (26.9)	0.48
Chronic congestive heart failure, n (%)	8 (8.6)	5 (9.6)	1.0
Hepatic failure, n (%)	2 (1.1)	1 (1.9)	1.0
Diabetes mellitus, n (%)	12 (12.9)	16 (30.8)	0.009

**Table 2:** Clinical parameters and biomarker differences evaluated at admission.

	AECOPD patients	CAP + AECOPD patients	*p*
No.	93	52	
Fever, n (%)	2 (2.2)	5 (9.6)	0.11
Increased sputum volume, n (%)	73 (78.5)	48 (92.3)	0.03
Aggravating cough, n (%)	89 (95.7)	50 (96.2)	1.0
Increased sputum purulence, n (%)	32 (34.4)	30 (57.7)	0.007
Pleuritic pain, n (%)	8 (8.6)	7 (13.5)	0.36
Dyspnea, n (%)	83 (89.2)	50 (96.2)	0.26
Intensive care unit admission, n (%)	2 (2.2)	2 (3.8)	0.95
Hospital stay, median (IQR)	8.0 (7.0–11.5)	10.0 (8.0–14.0)	0.03
Noninvasive mechanical ventilation, n (%)	2 (2.2)	3 (5.8)	0.50
Invasive mechanical ventilation, n (%)	0 (0)	0 (0)	-
Emergency treatment, n (%)	0 (0)	2 (3.8)	0.13
CRP, mg/dL	5.86 (3.27–18.7)	51.3 (16.6–102.7)	<0.001
PCT, ng/mL	0.049 (0.03–0.07)	0.09 (0.04–0.18)	<0.001
Neu (^*∗*^10^9/L)	4.68 (4.05–6.55)	6.78 (4.22–9.69)	0.01
EOS (^*∗*^10^9/L)	0.14 (0.02–0.35)	0.1 (0.02–0.19)	0.09
sTREM-1, pg/mL	115.3 (66–241.65)	104.3 (64.12–193.7)	0.47
OPN, ng/mL	12.61 (7.37–28.2)	20.6 (11.8–49.0)	0.015

**Table 3:** The area under the curve, sensitivity, specificity, predictive values, and cutoff values of CRP, PCT, Neu, and OPN.

Variable	AUC	Cutoff	Sensitivity (%)	Specificity (%)	Predictive values Positive	Negative
CRP	0.797	15.8 mg/dL	0.79	0.74	0.64 (47/74)	0.84 (80/95)
PCT	0.678	0.07 ng/mL	0.64	0.76	0.57 (39/68)	0.77 (78/101)
Neu	0.630	6.41*∗*10^9/L	0.54	0.75	0.56 (33/59)	0.74 (81/110)
OPN	0.622	13.26 ng/mL	0.73	0.53	0.47 (40/86)	0.73 (61/83)

**Table 4:** A logistic regression model for predicting CAP in AECOPD by clinical symptoms.

Variables	OR (95% CI)	*p* Value
Purulent sputum	2.39 (1.17–4.87)	0.02
Increased sputum volume	—	0.22
Diabetes mellitus	2.69 (1.13–6.39)	0.03
SCS treatment	—	0.18
FEV1, % predicted	—	0.13

Variables, including purulent sputum, increased sputum volume, diabetes mellitus, SCS treatment, and FEV1, % predicted, were retained as covariates in the forward stepwise selection procedure of multivariate analysis.

**Table 5:** Logistic regression model of factors predicting CAP in AECOPD.

Variables	OR (95% CI)	*p* Value
Purulent sputum	—	0.08
Increased sputum volume	—	0.19
Diabetes mellitus	—	0.06
SCS treatment	—	0.22
FEV1, % predicted	—	0.35
CRP ≥ 15.8 mg/dL	10.72 (4.76–24.13)	<0.001
Neu ≥ 6.41 × 10^9/L	—	0.16
PCT ≥ 0.07 ng/mL	—	0.10
OPN ≥ 13.26 ng/mL	—	0.26

Variables, including sputum purulence, increased sputum volume, diabetes mellitus, SCS treatment, FEV1, % predicted, PCT ≥ 0.07 ng/mL, Neu ≥ 6.41 × 10^9/L, CRP ≥ 15.8 mg/dL, and OPN ≥ 13.26 ng/mL, were retained as covariates in the forward stepwise selection procedure of multivariate analysis.

**Table 6:** The levels of CRP, PCT, Neu, EOS, sTREM-1, and OPN in patients with AECOPD/AECOPD + CAP according to the current use of ICS.

Variables	AECOPD			AECOPD + CAP		
	No ICS treatment	On ICS treatment	*p*	No ICS treatment	On ICS treatment	*p*
CRP (mg/dL)	7.0 (1.43–49.23)	5.5 (3.27–16.69)	0.92	18.85 (5.18–116.21)	51.83 (16.87–103.59)	0.57
Neu (×10^9/L)	5.06 (3.74–7.25)	4.66 (4.05–6.38)	0.74	4.25 (3.34–9.37)	6.9 (4.46–9.71)	0.45
EOS (×10^9/L)	0.04 (0.01–0.96)	0.15 (0.03–0.35)	0.60	0.12 (0.0–0.25)	0.1 (0.02–0.19)	0.86
PCT (ng/mL)	0.08 (0.04–0.13)	0.05 (0.03–0.07)	0.11	0.06 (0.04–0.0.32)	0.09 (0.04–0.18)	0.74
OPN (ng/mL)	18.3 (6.21–38.21)	12.55 (7.37–28.19)	0.84	17.32 (6.82–39.82)	21.21 (12.33–50.12)	0.41
sTREM-1 (pg/mL)	198.25 (77.04–404.44)	103.21 (62.37–227.3)	0.18	85.43 (56.53–212.98)	107.36 (63.88–204.04)	0.93

Notes: Values are expressed as median (interquartile ranges).

**Table 7:** The levels of CRP, PCT, Neu, EOS, sTREM-1, and OPN in patients with AECOPD/AECOPD + CAP according to the current use of SCS.

Variables	AECOPD			AECOPD + CAP		
	No steroid treatment	On steroid treatment	*p*	No steroid treatment	On steroid treatment	*p*
CRP (mg/dL)	5.50 (3.27–18.32)	8.62 (3.27–20.33)	0.57	60.68 (7.08–112.51)	47.65 (17.86–96.91)	0.91
Neu (×10^9/L)	4.59 (3.96–5.62)	5.43 (4.13–8.31)	0.04	4.98 (3.47–9.01)	7.16 (4.75–11.33)	0.04
Eos (×10^9/L)	0.16 (0.05–0.37)	0.07 (0.0–0.23)	0.01	0.11 (0.06–0.22)	0.05 (0.015–0.19)	0.26
PCT (ng/mL)	0.05 (0.03–0.07)	0.06 (0.03–0.11)	0.11	0.09 (0.04–0.21)	0.09 (0.05–0.16)	0.96
OPN (ng/mL)	12.42 (7.11–22.8)	12.85 (7.50–33.95)	0.28	23.81 (14.39–55.73)	20.22 (7.54–35.48)	0.25
sTREM-1 (pg/mL)	148.28 (71.9–272.12)	78.77 (40.9–115.68)	0.002	110.08 (63.88–228.80)	101.21 (57.21–156.17)	0.59

Notes: Values are expressed as median (interquartile ranges).

## Data Availability

The data that support the findings of this study are available from the corresponding author upon reasonable request.

## Abbreviations

COPD: Chronic obstructive pulmonary disease
AECOPD: Acute exacerbations of chronic obstructive pulmonary disease
AUC: Area under the curve
IQR: Interquartile range
CI: Confidence interval
ROC: Receiver operating characteristic
SD: Standard deviation
BMI: Body mass index
SCS: Systemic corticosteroids
ICS: Inhaled corticosteroids
FEV1/FVC: The ratio of the forced expiratory volume in the first 1 s to the forced vital capacity of the lungs
FEV1, % predicted: Baseline forced expiratory volume in the first 1 s, % of expected
GOLD: Global initiative for obstructive lung disease
ICU: Intensive care unit
CURB-65: Confusion, uremia, respiratory rate, blood pressure, age ≥65 years
CRP: C-reactive protein
PCT: Procalcitonin
Neu: Neutrophil
EOS: Eosinophil
sTREM-1: Soluble triggering receptor expressed on myeloid cells-1
OPN: Osteopontin
CAP: Community-acquired pneumonia
CT: Computed tomography.
